# Reduction of Uneven Brightness and Ghosts of Aerial Images Using a Prism in a Micromirror Array Plate

**DOI:** 10.3390/jimaging11030075

**Published:** 2025-03-03

**Authors:** Kaito Shoji, Yuto Osada, Atsutoshi Kurihara, Yue Bao

**Affiliations:** Graduate School of Integrative Science and Engineering, Tokyo City University, 1-28-1 Tamadutsumi, Tokyo 158-8557, Japan; g2081416@gmail.com (Y.O.); g2291401@tcu.ac.jp (A.K.); bao@tcu.ac.jp (Y.B.)

**Keywords:** aerial image, ghost removal, micromirror array plate, uneven luminance

## Abstract

A micro-mirror array plate is a type of aerial image display that allows an observer to touch the aerial image directly. The problem with this optical element is that it produces stray light, called a ghost, which reduces the visibility of the aerial image. Conventional methods can suppress the occurrence of ghosts; however, depending on the observation position, uneven luminance is produced in aerial images. Therefore, in this study, we proposed a method for eliminating ghosts while suppressing the unevenness in the luminance of an aerial image using a prism. In the proposed device, a prism is placed between the liquid crystal display and the diffuser, which is the light source of the aerial display. The experimental results showed that the proposed method can suppress the unevenness in the luminance of aerial images better than the conventional ghost removal methods and can reduce the formation of ghosts better than the micromirror array plate alone. Therefore, the proposed method can be shown to be a ghost removal method that can suppress unevenness in the brightness of aerial images.

## 1. Introduction

Aerial displays that can display images in empty air have gained significant research interest owing to their extraordinary nature [1,2]. In recent years, with the spread of coronavirus disease 2019, aerial displays have also attracted attention as noncontact tools for infection control [3]. Aerial displays are more hygienic than conventional touch panels because aerial images can be directly manipulated using sensors. For this reason, aerial displays are being developed in anticipation of increasing demand in restaurants, public facilities, medical institutions, and offices [4]. Two mainstream methods exist for aerial displays that display real images: aerial imaging by retroreflection (AIRR) [5] and transmissive mirror devices (TMDs) [6,7]. AIRR uses a retro-reflector and a beam splitter to form an image of a light source, such as a liquid crystal display (LCD), in mid-air; however, it requires a combination of two optical elements to display an image in mid-air but has the advantage of a wide viewing angle for the image in mid-air. By contrast, the TMD is an optical system that displays images in the air and only a TMD is required for aerial image formation. TMDs are primarily divided into two types: dihedral corner reflectors (DCRAs) and micromirror array plates (MMAPs). A DCRA [8,9,10] is an element on a plate composed of numerous corner reflectors and includes two main types: one is a plate with numerous square holes and the other is a group of corner cubes on the plate, with both types serving as corner reflectors. When light rays from a light source pass through the DCRA and are retroreflected by the corner reflectors inside the DCRA, they form an aerial image. In this case, the aerial image is formed at a position that is plane-symmetrical to the light source with respect to the DCRA. The user can observe aerial images without wearing glasses or other devices. The MMAP used in this study [11,12,13,14] is an optical element consisting of two layers of orthogonal mirror arrays and operates in a manner similar to the DCRA. In the MMAP, light from a light source is reflected an odd number of times from each layer to form an aerial image. However, when such reflections do not occur, light rays may pass through the MMAP or stray light, called a ghost, may be generated. Ghosts can cause images to appear in unintended positions, leading to confusion. In addition, when ghosts overlap, the visibility of the aerial image is reduced [15]. To address this problem, a method for blocking light rays that cause ghosting was proposed [7]. However, this method reduces the luminance of the aerial image. Consequently, a method was proposed to control the formation of ghost rays in an aerial image [14]. Although this method improves the luminance of the aerial image, the luminance is uneven depending on the observation position. Therefore, in this study, we proposed a method for reducing the formation of ghosts while minimizing the unevenness in the luminance of aerial images. By reducing luminance irregularities, it is expected to display aerial images with constant luminance without adjusting the luminance according to the observation position.

## 2. Conventional Method

### 2.1. Principle of the MMAP

**Figure 1:**
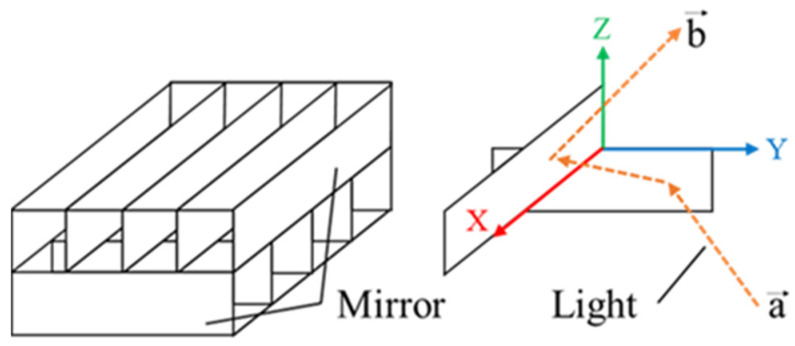
Principle of the MMAP.

Figure 1 shows the structure of the MMAP, which consists of two layers of orthogonal mirror arrays.

The incident light is reflected once from each layer of the MMAP to form an aerial image at a position that is plane-symmetrical to the MMAP. The mirror surfaces of each layer are arranged such that they face the positive directions of the x- and y-axes. As shown in Figure 1, the twice-reflected incident vector “a” and the ray vector “b” can be expressed using (1) and (2). Equations (1) and (2) show that, in the MMAP, as in the DCRA, the light rays forming the aerial image are retroreflected when they pass through the interior of the element.

(1) $$\vec{a}=\left(a_{x},a_{y},a_{z}\right)$$

(2) $$\vec{b}=\left({-a}_{x},{-a}_{y},a_{z}\right)$$

Furthermore, as summarized in Table 1, the image differs depending on the number of reflections in each layer of the MMAP [15].

When the light rays emitted from a light source are reflected an odd number of times in each layer, they are focused as an aerial image at a position that is plane-symmetric with respect to the element. However, if one layer reflects an odd number of reflections and the other layer reflects an even number of reflections, the reflection is specular. This image is called a ghost image. The number of reflections in an element depends on the angle of incidence of light from the light source.

### 2.2. Method of Ghost Reduction

**Figure 2:**
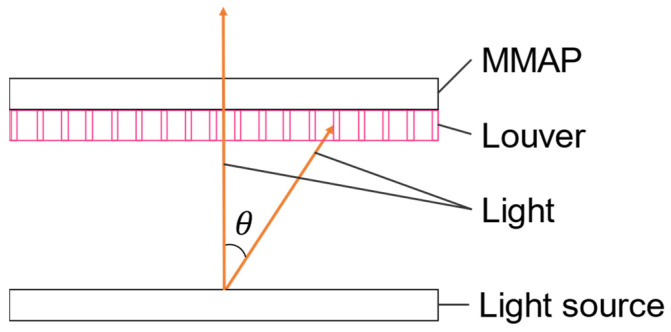
Principle of removing ghosts using the conventional method with a louver.

One method of removing ghosts while displaying aerial images is to use louvers (viewing-angle control films) [7]. Figure 2 shows the configuration of the ghost removal method using a louver.

Louvers block light rays outside of a certain range and transmit the light rays within that range. Ghosts can be removed by limiting the angle of incidence to the MMAP to 30° or less; however, this also removes the light rays that form the aerial image, thus limiting the viewing angle of the aerial image and reducing its brightness depending on the film [7,13].

**Figure 3:**
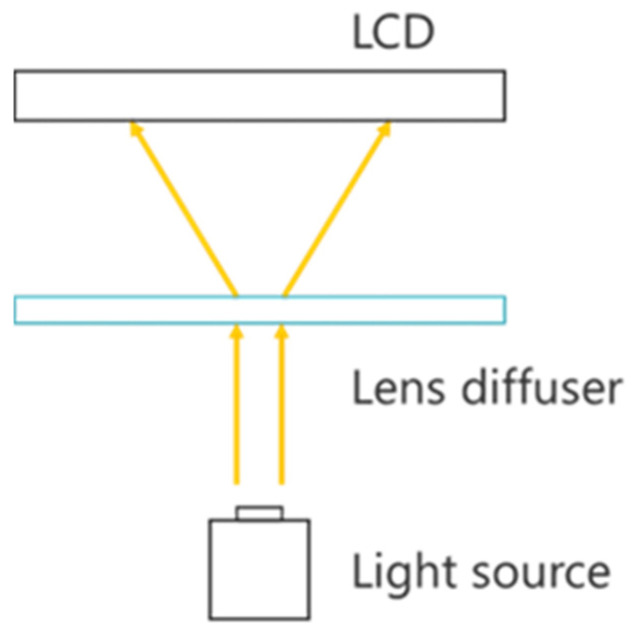
Principle of removing ghosts using the conventional method with a lens diffuser.

To address this problem, we previously proposed a ghost removal method using a lens diffuser [14]. In this method, the light rays that form ghosts are not removed but are instead formed as an aerial image, thereby removing the ghosts and improving the brightness of the aerial image. A lens diffuser limits the diffusion of light to a specific range using the diffusion function of a minute, random lens array [16]. A lens diffuser diffuses light within a certain range centered at a position with a normal direction of 0° and is designed such that light rays with an angle of incidence of 0° have the highest luminance. Figure 3 shows the principle of the ghost elimination method using a lens diffuser, in which light from the light source diffused by the lens diffuser is transmitted to the LCD.

The angle of incidence of light in the MMAP is equal to the angle of exit of light diffused by the lens diffuser. Therefore, ghosting can be eliminated by maintaining the exit angle of the light diffused by the lens diffuser within a range that does not contribute to ghost formation. In addition, by focusing the light with a lens diffuser, the light intensity is increased and, consequently, the brightness of the aerial image is improved. However, because the lens diffuser diffuses light such that the light rays at 0° are at their maximum, there is a problem of uneven luminance in the aerial image depending on the observation position.

## 3. Proposed Method

### 3.1. Principle of the Proposed Method

In the method that uses a louver to remove specific light rays, the aerial image becomes darker; therefore, the light from the light source must be stronger. In addition, the method of using a lens diffuser to form a ghost ray as an aerial image improves the luminance of the aerial image; however, it is difficult to display the aerial image at a luminance suitable for the observer because uneven luminance is noticeable depending on the observation position. To overcome these limitations, in this paper, we proposed a method that forms a ghost ray as an aerial image and suppresses the unevenness in the luminance of the aerial image.

**Figure 4:**
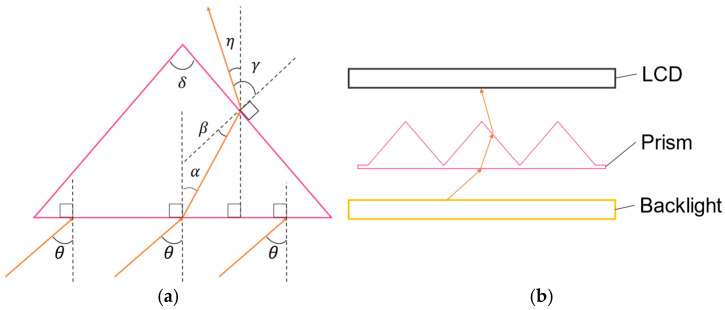
Principle of the proposed method. (**a**) State of refraction of light rays. (**b**) Configuration of the proposed device.

Previously, we focused on the refraction of light by prisms and proposed a ghost removal method using MMAP. In this study, we propose a prism-based method for reducing luminance irregularities in aerial images. The configuration of the proposed system is shown in Figure 4a. Figure 4b shows the state of light refraction by the prism.

In the proposed device, a prism is placed between the LCD and the LCD backlight, which is the light source of the aerial display. Because light is refracted at the boundaries between media with different refractive indices, an appropriate prism can refract the light rays that form ghost and aerial images. Although prisms can refract light to make it more directional, they do not focus light as strongly at the center as a lens diffuser. Therefore, since the viewing angle of the MMAP aerial image is 20° to the left and right [4], we thought that uneven luminance of the aerial image could be suppressed if the luminance distribution of the prism within that range was not such that it emphasized the peak of the luminance distribution of the light source.

As shown in Figure 4a, let θ be the angle of incidence to the flat side of the prism, α be the angle of emission from the flat side of the prism, β be the angle of incidence to the apex of the prism, γ be the angle of emission from the apex of the prism, and η be the angle of deviation of the emitted light from the vertical direction. The refractive index of air is 1 and that of the prism is n. The amount of incident light that is refracted is calculated based on Snell’s law [17] using the following Equation:

(3) $$\alpha =arc\sin\frac{\sin\theta }{n}$$

(4) $$\beta =\frac{\pi -\delta }{2}-\alpha$$

(5) $$\gamma =arc\sin\left(n\sin\beta \right)$$

(6) $$\eta =\gamma -\frac{\pi -\delta }{2}$$

In the conventional method, the incident angle of light into the MMAP is blocked or refracted such that it falls within 30°. Therefore, the angle of incidence of light rays into the prism was changed to calculate the angle of exit and a prism whose angle of exit is within 30° to the left and right was selected.

### 3.2. Simulation of the Prism

**Figure 5:**
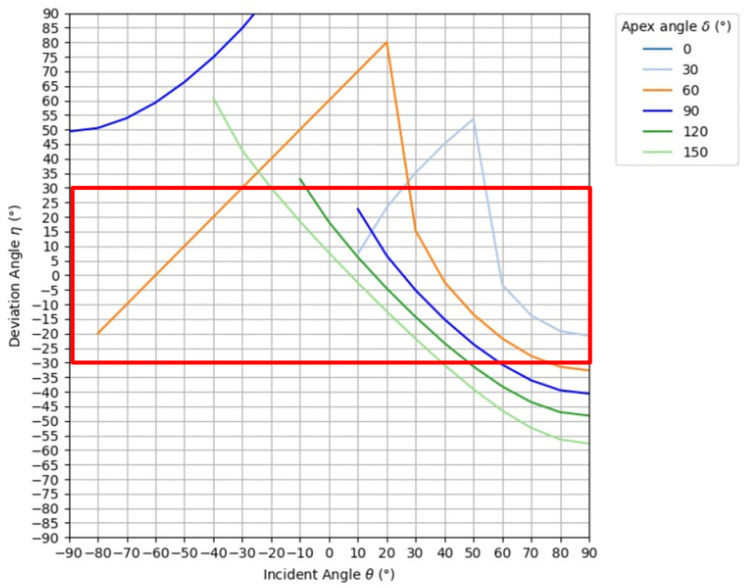
Results of refraction simulation on the right slope of a prism.

In this study, the optimal apex angle was investigated using polymethylmethacrylate (PMMA) as the optical material because PMMA is transparent and has a small dispersion comparable to that of optical glass [18]. The refractive index of PMMA is 1.492 at the sodium D line (589.3 nm), which is the reference wavelength. Figure 5 shows the results of refraction simulations in which the apex angle was varied from 0° to 165° in 15° increments and the angle of incidence was varied from 0° to 90° in 10° increments. Here, the red frame in the graph shows the range of outgoing light from −30° to 30°, which is less likely to contribute to the formation of ghosts. Also, this simulation considers only rays incident on the right slope of the prism, and the simulation results for rays incident on the left slope are inverted vertically and horizontally with respect to the origin. Here, if total reflection occurs at the first prism face, only the light immediately emitted from the other prism face is shown, and no more than two re-reflections are considered.

Figure 5 shows that, when the apex angle ranges between 30° and 90°, total reflection occurs once at the prism face and the second refraction at the prism face removes light from the 30° range. A vertex angle of 120° or more is refracted the first time on the prism surface; however, as the angle of incidence increases, the deviation angle falls outside the 30° range. Therefore, regardless of the apex angle, the light rays at 30° to the left and right, which tend to contribute to the ghosts, cannot be eliminated. Therefore, we next consider the luminance of the rays of light emitted from the prism. The light rays incident on the prism are partially reflected and lost at the bottom of the prism, and either refracted or reflected at the slopes. Furthermore, considering the ratio of rays incident on each slope, the luminance of rays emitted from the prism can be shown by Equations (7) and (8) for refraction and reflection, respectively.

(7) $$L_{o}=L_{i}\times T_{b}\times T_{r}\times R_{s}$$

(8) $$L_{o}=L_{i}\times T_{b}\times (1-T_{r})\times R_{s}$$

where $L_{i}$ is the luminance of the incident light, $T_{b}$ is the transmittance at the bottom of the prism, $T_{i}$ is the transmittance at the slope (ratio of refraction), $R_{s}$ is the ratio of rays incident on the right (or left) slope, and $L_{o}$ is the luminance of the rays emitted from the prism. By performing this calculation for each slope of the prism, the luminance of the rays emitted from the prism can be determined. Here, the transmittance of light rays passing through the prism can be determined by using p-polarized light and s-polarized light. The terms p-polarized light and s-polarized light refer to the type of polarization that occurs when light is reflected or refracted with respect to the surface of an object. Polarization in which light oscillates parallel to the plane of incidence is called p-polarized light, while polarization in which light oscillates perpendicular to the plane of incidence is called s-polarized light. Let the angle of incidence to the object be $\theta_{i}$ and the angle of refraction be $\theta_{t}$, and let the refractive indices of the medium before and after transmission be $n_{1}$ and $n_{2}$, respectively, the amplitude transmission coefficients for each polarized light $t_{p}$, $t_{s}$ can be expressed by Equations (9) and (10), and these can be used to obtain the transmittance $T_{p}$, $T_{s}$ for each polarization as in Equations (11) and (12) [19]. Considering the ratio of p-polarized light to s-polarized light as 1 to 1, the transmittance T to the prism can be expressed by Equation (13).

(9) $$t_{p}=\frac{2n_{1}cos\theta_{i}}{n_{2}cos\theta_{i}+n_{1}cos\theta_{t}}$$

(10) $$t_{s}=\frac{2n_{1}cos\theta_{i}}{n_{1}cos\theta_{i}+n_{2}cos\theta_{t}}$$

(11) $$T_{p}=\left(\frac{n_{2}cos\theta_{t}}{n_{1}cos\theta_{i}}\right){\left|t_{p}\right|}^{2}$$

(12) $$T_{s}=\left(\frac{n_{2}cos\theta_{t}}{n_{1}cos\theta_{i}}\right){\left|t_{s}\right|}^{2}$$

(13) $$T={0.5T}_{p}+{0.5T}_{s}$$

**Figure 6:**
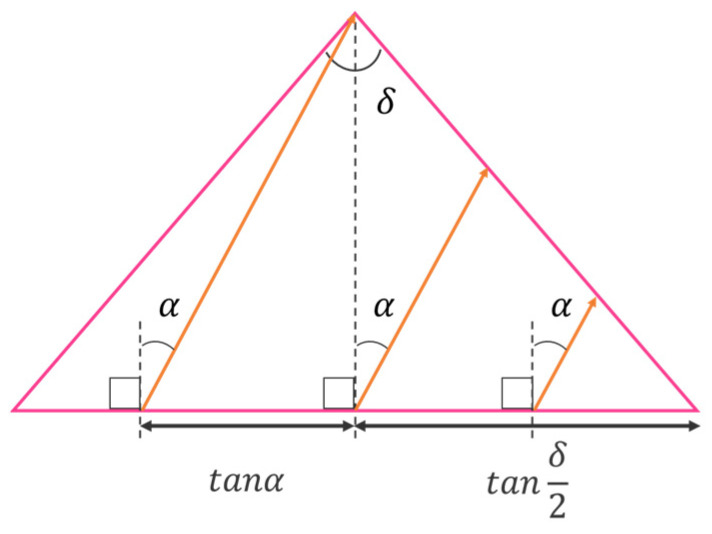
Ratio of rays incident on the right slope of the prism.

Figure 6 shows rays of light incident on the right slope of the prism. As shown in Figure 6, the rays incident on each slope fall within a certain range. Therefore, the proportion of rays entering each slope $R_{s}$ is shown in Equation (14).

(14) $$R_{s}=\frac{tan\frac{\delta }{2}+tan\alpha }{2tan\frac{\delta }{2}}$$

**Figure 7:**
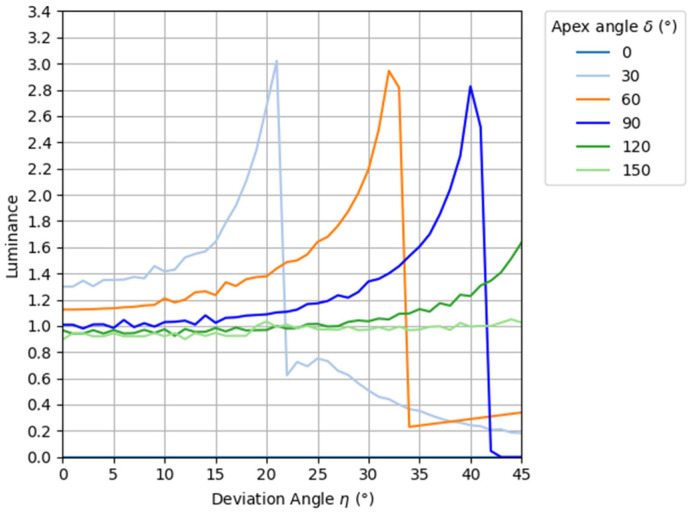
Results of luminance simulation.

From the above Equations (7) to (14), the simulation results of the luminance of light passing through the prism at each angle can be shown in Figure 7. Here, the simulation was performed over the same range of apex angles as in Figure 5, and the luminance of light rays incident on the prism was set to 1 regardless of the angle. The vertical axis represents the luminance of rays emitted from the prism, and the horizontal axis represents the angle of exit from the prism.

**Figure 8:**
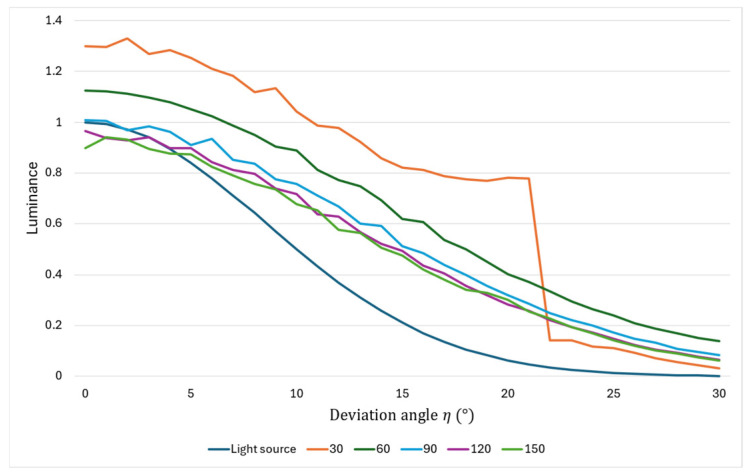
Luminance distribution of light source and prism combined.

As shown in Figure 7, the brightness of the prism improves as the declination increases within a viewing angle of 20° of the aerial image. Therefore, the luminance distribution is not in the shape of a normal distribution as in the louver and lens diffusion of conventional methods, and the peak of the luminance distribution of the light source is not emphasized. In other words, it is thought that the use of prisms can lead to the reduction of uneven luminance of aerial images. Here, considering the angular range of incident light on the MMAP, which tends to contribute to ghosting, we consider a normally distributed light source whose luminance is zero at 30°. The superimposed distribution of this light source and prism is shown in Figure 8.

From Figure 8, it can be seen that there is a significant increase or decrease in luminance in some angular ranges when the apex angle is 30°, but when prisms with other apex angles are used, we can see that the distribution is more gradual than that of light sources alone. Therefore, the use of a prism with a vertex angle between 60° and 150° can be expected to reduce the luminance irregularity of the aerial image, and considering the luminance efficiency, a prism with a vertex angle of 60° is considered to be the most suitable for this method.

## 4. Experiments and Results

To verify the effectiveness of the proposed method, experiments were conducted to measure the luminance of aerial images and ghosts for each method. Details of the experimental setup are listed in Table 2. Based on the simulation results, the optimal prism was the one with a 60° apex angle; however, in this experiment, a prism with a 90° apex angle, which is relatively easy to obtain, was used. This prism is not optimal for ghost removal but is suitable for reducing the uneven brightness of aerial images. The thickness of the normal prism increases proportionally to the size of the light source. Therefore, a prism array was used in the experiments. The louvers were selected from those recommended by Asukanet Co. Ltd. (Hiroshima, Japan), which sold the MMAPs used in this study.

### 4.1. Determining the Relationship Between the Luminance of Light Sources and the Camera Pixel Values

**Figure 9:**
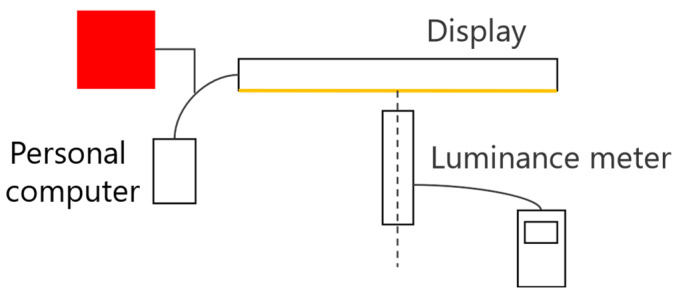
Measuring the display luminance with a luminance meter.

**Figure 10:**
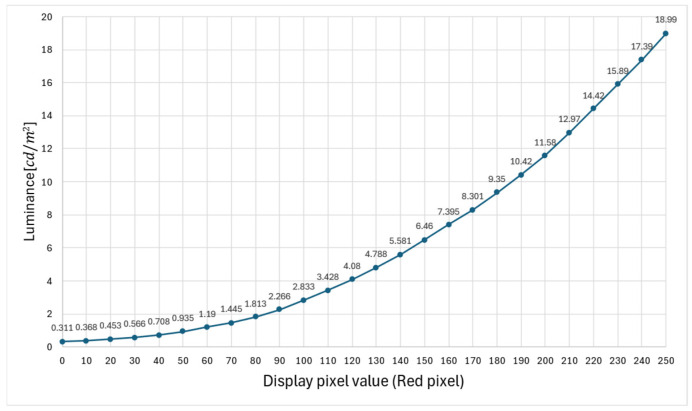
Relationship between the pixel value and the brightness of the display.

The luminance meter owned by the authors could not directly measure the luminance of an aerial image because the luminance must be measured by contacting the object to be measured. Therefore, we considered the camera as a pseudo-luminance meter. By measuring the relationship between the luminance value of the display, which is the light source, and the pixel value of the image, the luminance can be calculated from the pixel value of the image captured by the camera. In a previous study, we conducted experiments with red pixels, with the display luminance set to the maximum and contrast set to standard values, with pixel values ranging from 0 to 250 in increments of 10. Figure 9 shows the arrangement of the experimental apparatus used to measure the luminance of the display when viewed from the vertical direction. Because the display used for the measurements was the same as that used in a previous study, the measurement data recorded in the previous study were used in the present study [14]. The measurement results are shown in Figure 10.

#### 4.1.1. Calculation of Luminance in Camera-Captured Images

**Figure 11:**
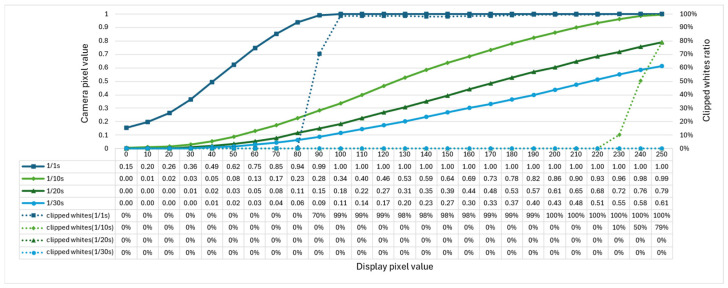
Relationship between the pixel values of the camera and display.

Because the pixel values of acquired images could not be accurately determined owing to the presence of whiteouts depending on the luminance of aerial images, the camera shutter speeds were set to 1/1 s, 1/10 s, 1/20 s, and 1/30 s. Because the images were captured in a dark room, the aperture was set to f/3.5 and the ISO sensitivity was fixed at 100, which is the lowest value available. The results are presented in Figure 11. The vertical axis in Figure 11 shows the pixel values of the captured images normalized from 0 to 1, and the horizontal axis shows the red pixel values on the display.

The results shown in Figure 11 confirm that there is no white skipping in the range of display pixel values from 0 to 80 for a shutter speed of 1/1 s, from 0 to 220 for 1/10 s, and in any range for 1/20 s and 1/30 s. Using the no-whiteout range as a target, we approximated the range of pixel values of the displayed pixels captured at each shutter speed to a polynomial of the fourth degree using the least-squares method [14]. If the luminance value is y and the camera pixel value normalized to 0–1 is x, the approximate Equations are expressed in (15) to (18).

(15) $$y_{1/1s}=2.923x^{4}-3.460x^{3}+1.660x^{2}+0.825x+0.162$$

(16) $$y_{1/10s}=18.033x^{4}-16.128x^{3}+9.906x^{2}+5.065x+0.364$$

(17) $$y_{1/20s}=18.380x^{4}-4.710x^{3}+6.910x^{2}+12.003x+0.418$$

(18) $$y_{1/30s}=2.979x^{4}+18.596x^{3}-3.268x^{2}+20.207x+0.485$$

**Figure 12:**
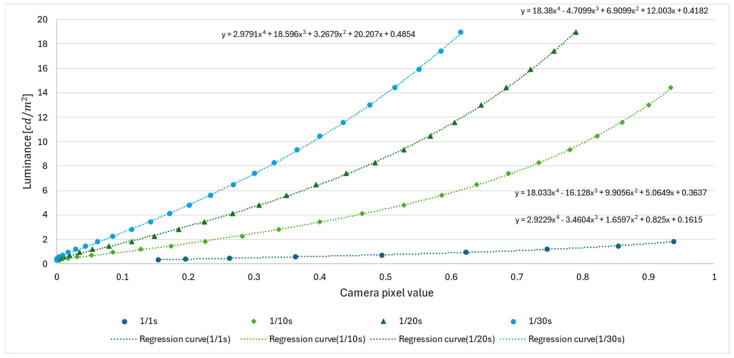
Relationship between the pixel values of the camera and the luminance of the luminance meter.

Figure 12 summarizes the results of the relationship between the pixel values of the camera and the luminance values of the luminance system for each shutter speed.

#### 4.1.2. Experiments on Capturing Aerial Images

**Figure 13:**
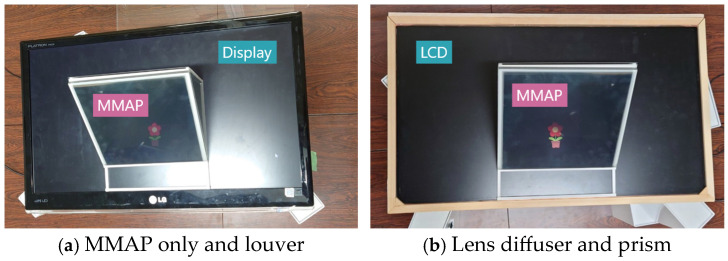
Experimental setup.

**Figure 14:**
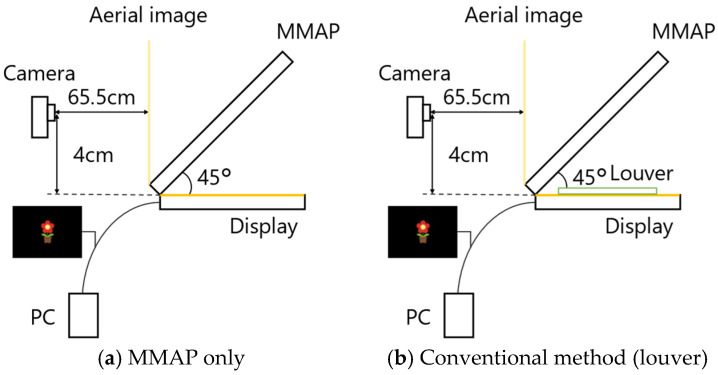

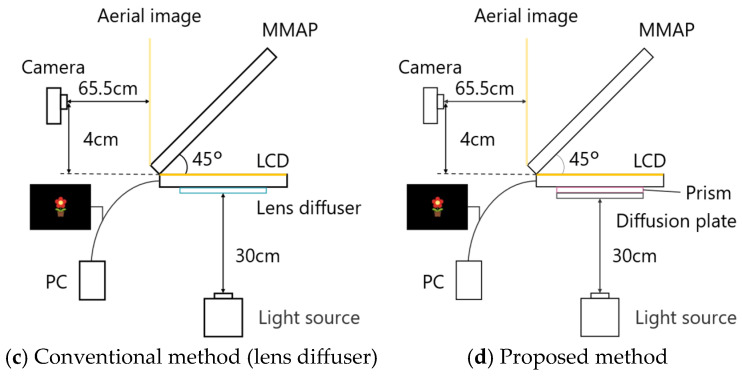
Configuration of experimental setup for each method (side view).

To verify the effectiveness of the proposed method, we compared the brightness of the aerial images and ghosts of the MMAP only, the conventional method with louvers and lens diffusers, and the proposed method. Figure 13 shows the experimental setups used for MMAP only and louvers, and for lens diffusers and prisms. Figure 14 shows the experimental setup for each method viewed from the side.

**Figure 15:**
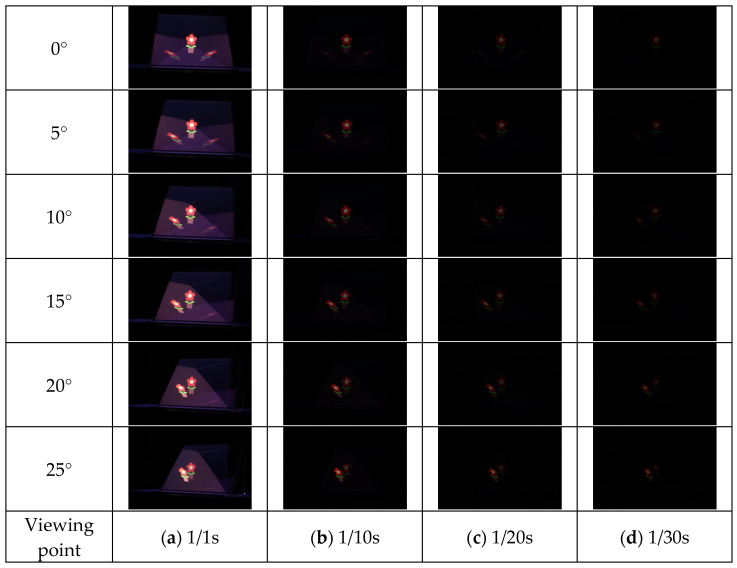
Shooting results (MMAP only).

**Figure 16:**
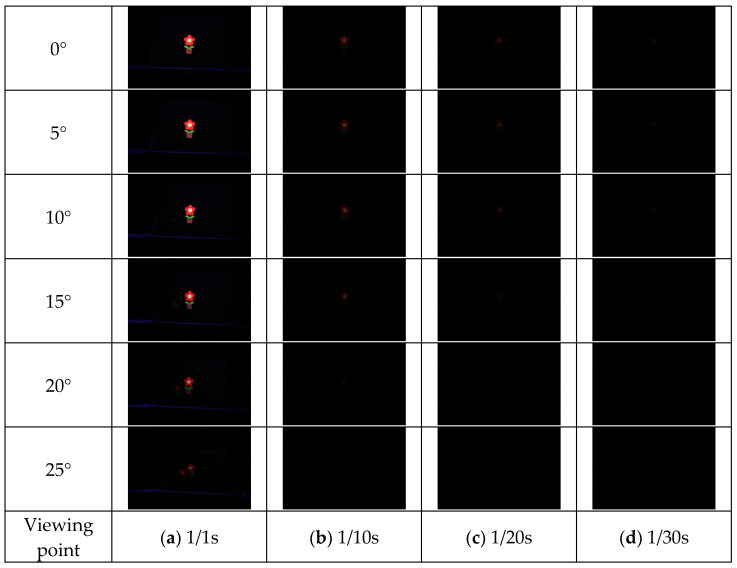
Shooting results (louver).

**Figure 17:**
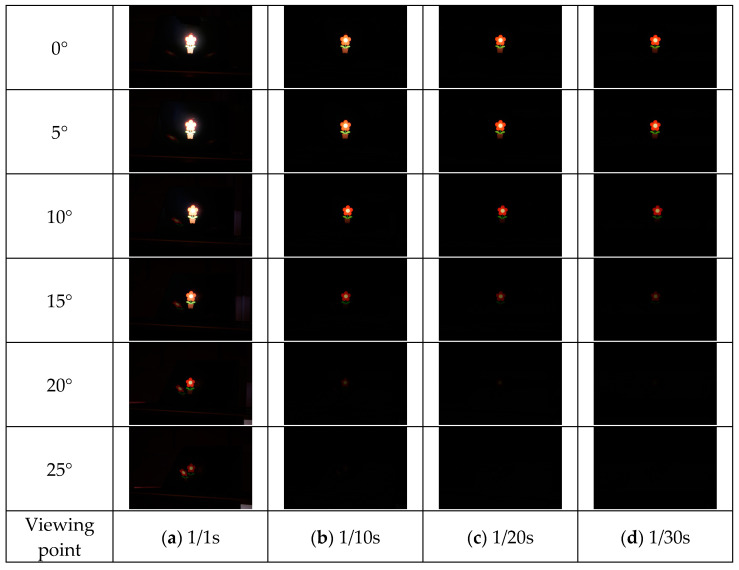
Shooting results (lens diffuser).

**Figure 18:**
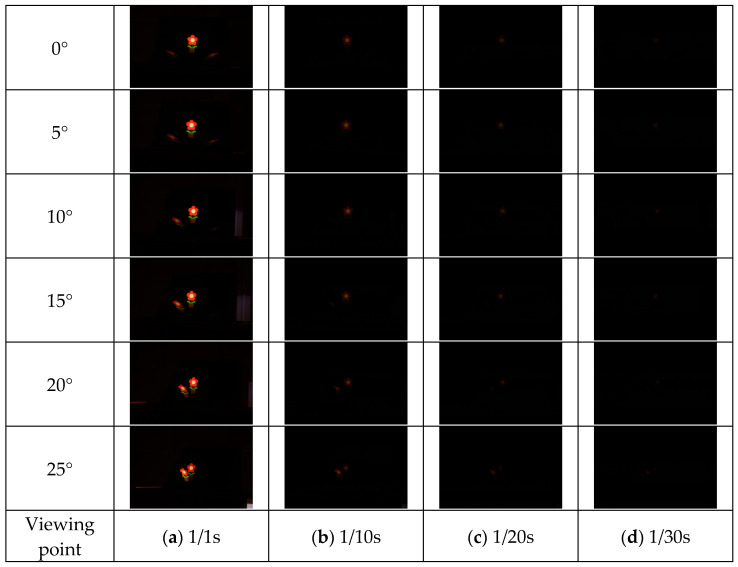
Shooting results (prism).

The results of each method are shown in Figure 15, Figure 16, Figure 17 and Figure 18. Here, the camera shutter speeds were set to 1/1 s, 1/10 s, 1/20 s, and 1/30 s to suppress the effects of ghost blackouts and whiteouts of the aerial image. In addition, because the MMAP used had a viewing angle of 20° from left to right, images were captured in 5° increments from 0° to 25° horizontally.

To calculate the brightness of each image, a portion of the petal of a flower was cut, and its average pixel value was used. The pixel values were normalized to fall within the range of 0 to 1. The pixel values for each method are listed in Table 3, Table 4, Table 5 and Table 6. The estimated luminance values for each method were calculated using the pixel values and approximate formulas expressed in (7)–(10). The average of the estimated luminance values calculated at each shutter speed was used as the estimated luminance value for each method. However, to suppress the effects of whiteout and blackout, pixel values of 0.98 or higher and those of 0.02 or lower were excluded from the calculations [14]. In these tables, the data used in the calculations are indicated in bold.

**Figure 19:**
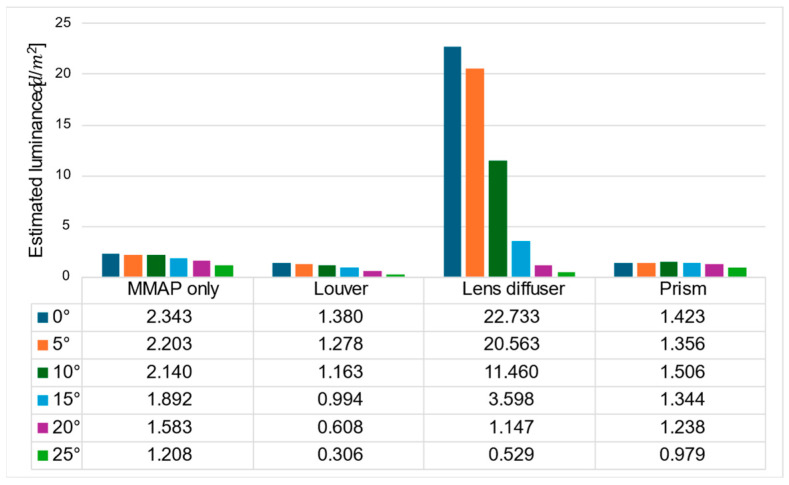
Estimated luminance of aerial images for each method.

**Figure 20:**
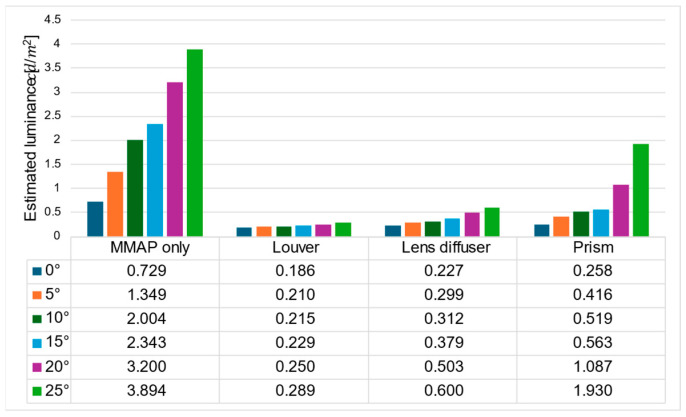
Estimated luminance of ghosts for each method.

Figure 19 and Figure 20 show the estimated luminance values for each angle of the aerial image and the ghost for each method, respectively.

**Figure 21:**
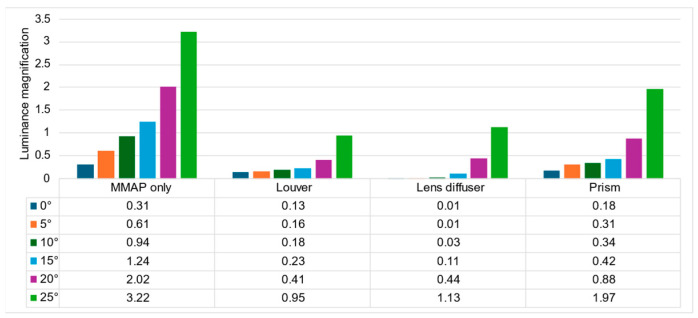
Luminance ratio of ghost to aerial image.

The ratio of the luminance of the ghost to that of the aerial image is shown in Figure 20, and the ratio of the luminance of the aerial image at adjacent angles is shown in Figure 21.

**Figure 22:**
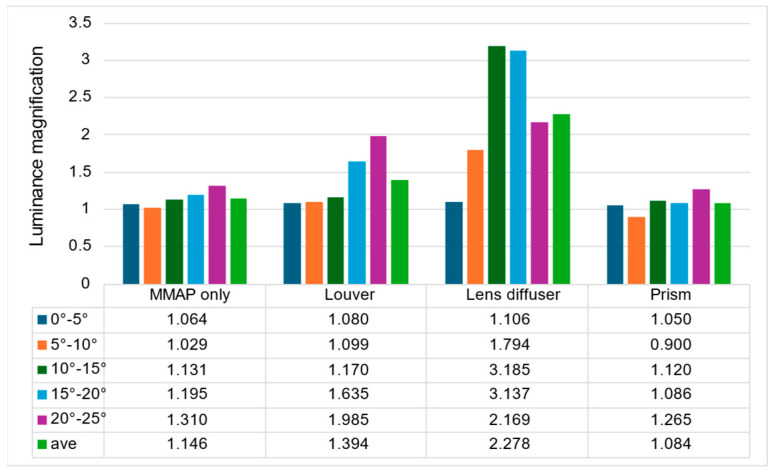
Luminance ratio of aerial images at adjacent angles.

Figure 19 shows that the prism-based method produces a higher luminance for the aerial image at all angles than the louver-based method. This may be because a louver blocks light as the angle increases, but the prism does not; therefore, the luminance does not decrease significantly. In addition, the luminance of the aerial image with the prism is lower than that of the aerial image with the lens diffuser from 0° to 15° and higher at 20° and 25°, which is considered to be because the prism emits light over a wider range than the lens diffuser. Figure 21 shows that the prism-based method eliminates ghosts better than MMAP alone and has the highest luminance of all ghost elimination methods. This is because the prism-based method cannot remove rays at 30° or more to the left or right, which are more likely to cause ghosting. Figure 22 shows that the prism method reduces the average luminance ratio of the aerial image by 6.2%, 31.0%, and 119.4% compared with MMAP-alone, louver, and lens diffuser methods, respectively. Therefore, we confirmed that the proposed method could reduce the luminance irregularity of aerial images better than the conventional ghost removal methods. Figure 19 also shows that luminance irregularities also occur in the case of MMAP alone. This suggests that angle-dependent luminance irregularities may exist in MMAP as well, and we would like to address this issue in the future.

## 5. Conclusions

In this study, we focused on the problem of ghosting that occurs when displaying aerial images on the MMAP and the problem of uneven luminance of aerial images that occurs in conventional ghost removal methods. We attempted to eliminate ghosting by placing a prism between the LCD and the diffuser, which has little effect on the luminance distribution of the light source, to suppress the uneven luminance of the aerial image and make the display more directional. To confirm the effectiveness of the proposed method, luminance values of the aerial image and ghost were measured and compared for each method. The proposed method reduced luminance irregularities better than the other ghost removal methods, and the method using a prism was the most effective in reducing luminance irregularities. The proposed method reduced ghosting more than MMAP alone, although it produced a higher luminance than the other ghost removal methods. Therefore, the results demonstrate that the proposed method can reduce luminance irregularities and eliminate ghosts in aerial images.

## Figures and Tables

**Table 1:** Relationship between images and number of reflections by the MMAP.

		First Layer	
		Odd Times	Even Times
Second layer	Odd times	Aerial image	Ghost
	Even times	Ghost	Transmitted light

**Table 2:** Details of experimental equipment.

Equipment	Parameters	Specification
MMAP	model number	ASKA3D-200NT
	size	200 × 200 mm
	pitch	0.3 mm
	viewing angle	40°
	material	Optical Resins
Display, LCD	model number	LG Electronics IPS236V
	resolution	1920 × 1080 pixels (23 inch) Used 125 × 125 mm inside
LED	model number	OHM LED E26 L70313
Prism array	model number	LPV-200S-90-0.05
	size	200 × 200 × 2 mm
	apex angle	90°
	prism pitch	0.05 mm
	material	PMMA
	index of refraction	1.492
Diffusion plate	Disassembly source	IBM T221TFT LCD
Lens diffuser	model number	LSD10PC10-5
	diffusion angle [deg]	10
	size	5.0 × 5.0 inch
	material	polycarbonate
Louver	model number	Hikari kogyo Looknon-N8
	viewing angle	60°
	visible light transmittance	71.2%
	material	PET
Camera	model number	SONY α6000
	lens	E 30mm F3.5 Macro

**Table 3:** Results of pixel values (MMAP only).

			Aerial Image			Ghost		
	1/1 s	1/10 s	1/20 s	1/30 s	1/1 s	1/10 s	1/20 s	1/30 s
0°	0.994	**0.285**	**0.149**	**0.089**	**0.534**	**0.059**	**0.024**	0.011
5°	**0.975**	**0.268**	**0.138**	**0.083**	**0.829**	**0.160**	**0.073**	**0.040**
10°	**0.974**	**0.266**	**0.141**	**0.081**	**0.958**	**0.249**	**0.128**	**0.074**
15°	**0.936**	**0.233**	**0.120**	**0.068**	0.994	**0.290**	**0.157**	**0.094**
20°	**0.882**	**0.191**	**0.093**	**0.053**	0.999	**0.409**	**0.231**	**0.150**
25°	**0.772**	**0.141**	**0.061**	**0.034**	1.000	**0.498**	**0.288**	**0.189**

**Table 4:** Results of pixel values (louver).

			Aerial Image			Ghost		
	1/1 s	1/10 s	1/20 s	1/30 s	1/1 s	1/10 s	1/20 s	1/30 s
0°	**0.845**	**0.162**	**0.073**	**0.042**	**0.028**	0.001	0.000	0.000
5°	**0.809**	**0.148**	**0.067**	**0.037**	**0.053**	0.001	0.000	0.000
10°	**0.767**	**0.130**	**0.058**	**0.032**	**0.059**	0.001	0.000	0.000
15°	**0.694**	**0.105**	**0.044**	**0.024**	**0.073**	0.002	0.001	0.000
20°	**0.419**	**0.040**	0.016	0.008	**0.093**	0.003	0.001	0.000
25°	**0.144**	0.006	0.002	0.001	**0.129**	0.005	0.001	0.001

**Table 5:** Results of pixel values (lens diffuser).

			Aerial Image			Ghost		
	1/1 s	1/10 s	1/20 s	1/30 s	1/1 s	1/10 s	1/20 s	1/30 s
0°	0.998	1.000	0.999	**0.923**	**0.071**	0.002	0.001	0.000
5°	0.998	1.000	**0.970**	**0.842**	**0.138**	0.005	0.002	0.001
10°	1.000	**0.921**	**0.690**	**0.535**	**0.149**	0.007	0.002	0.001
15°	1.000	**0.457**	**0.264**	**0.174**	**0.208**	0.013	0.004	0.002
20°	**0.759**	**0.130**	**0.057**	**0.030**	**0.315**	**0.025**	0.009	0.004
25°	**0.342**	**0.029**	0.011	0.006	**0.416**	**0.038**	0.015	0.007

**Table 6:** Results of pixel values (Prism).

			Aerial image			Ghost		
	1/1 s	1/10 s	1/20 s	1/30 s	1/1 s	1/10 s	1/20 s	1/30 s
0°	**0.859**	**0.168**	**0.078**	**0.044**	**0.100**	0.004	0.001	0.000
5°	**0.843**	**0.159**	**0.072**	**0.040**	**0.239**	0.017	0.005	0.003
10°	**0.881**	**0.180**	**0.082**	**0.049**	**0.334**	**0.027**	0.010	0.004
15°	**0.834**	**0.158**	**0.071**	**0.040**	**0.381**	**0.033**	0.012	0.006
20°	**0.801**	**0.142**	**0.063**	**0.035**	**0.726**	**0.118**	**0.052**	**0.029**
25°	**0.859**	**0.168**	**0.078**	**0.044**	**0.100**	0.004	0.001	0.000

## Data Availability

The raw data supporting the conclusions of this article will be made available by the authors on request.
